# A new method to quantify the human visual threshold from melanopsin sensitive ganglion cells

**DOI:** 10.3389/fncel.2023.1132230

**Published:** 2023-03-23

**Authors:** Jeff Rabin, Erica Poole, William Price, Gurjiv Kaur, Kiana Hall, Venessa Sailors, Brazil Andrews, Rathanart Somphruek

**Affiliations:** Rosenberg School of Optometry, University of the Incarnate Word, San Antonio, TX, United States

**Keywords:** melanopsin, ganglion cells, intrinsically photosensitive retinal ganglion cells (ipRGCs), visual perception, pupil

## Abstract

Traditional photoreceptors utilize the chromophore retinal to absorb light coupled with a unique opsin protein to specify receptor spectral sensitivity. Light absorption triggers a cascade of events transducing light energy to neural signals beginning with graded potentials in receptors (rods and cones) and bipolar cells in outer and middle retina eventuating in action potentials at the inner retinal amacrine and ganglion cell levels. Unlike traditional photoreceptors, ganglion cells in the inner retina (intrinsically photosensitive retinal ganglion cells, ipRGCs) absorb short wavelength, blue light utilizing their photopigment melanopsin. Assessment across multiple species show that the ipRGCs mediate myriad visual and non-visual functions including photo-entrainment and circadian rhythms, the pupillary light reflex, sleep, alertness, cognition, mood, and even conscious visual perception. Some ipRGC functions can persist despite blindness in animal models and humans exemplifying their multidisciplinary control of visual and non-visual functions. In previous research we used selective chromatic adaptation (blue stimulus on a bright amber field) to suppress input from rods, red and green sensitive cones to identify retinal and cortical responses from ipRGCs. Herein we used a similar approach, coupled with a filter to block input from blue sensitive cones, to develop a clinically expedient method to measure the full-field, putative visual threshold from human ipRGCs. This metric may expand our ability to detect, diagnose and monitor ocular and neurologic disease and provide a global retinal metric of ipRGCs as a potential outcome measure for studies using gene therapy to arrest and/or improve vision in hereditary retinal diseases.

## Introduction

Intrinsically photosensitive retinal ganglion cells (ipRGCs) absorb short wavelength, blue light utilizing their inherent photopigment: melanopsin (Berson et al., 2002; Hattar et al., 2002; Do, 2019; Aranda and Schmidt, 2020; Mure, 2021). After their discovery some 20 years ago (Hattar et al., 2002; Mure, 2021) these *fifth* retinal receptors in the human eye have been identified in multiple species with mouse and sub-human primates being the most studied with definitive invasive techniques (Aranda and Schmidt, 2020). Mouse studies reveal a broad diversity of ipRGCs (Sondereker et al., 2020) and studies of non-human primates have expanded our understanding of ipRGCs in visual systems comparable to the human visual system (Dacey et al., 2005). There is now multiple evidence that ipRGCs mediate a plethora of visual and non-visual functions despite their small proportion of ganglion cells (up to 7,000 in humans vs. 1 million ganglion cells overall, 0.7%). Yet these cells are diverse in their anatomical size, diversity, physiological responses, neurologic connectivity, and possibly phylogenetic origins (Dacey et al., 2005; Do, 2019; Abbas et al., 2020; Sondereker et al., 2020). ipRGCs convey action potentials to various neural sites which impact photo-entrainment, circadian rhythms, the pupil light reflex (PLR), sleep, alertness, cognition, mood as well as conscious visual perception (Berson et al., 2002; Hattar et al., 2002; Dacey et al., 2005; Do, 2019; Mure et al., 2019; Abbas et al., 2020; Aranda and Schmidt, 2020; Sondereker et al., 2020; Mure, 2021). Functions including photo-entrainment and PLR can persist despite blindness in animal models and humans with blinding disease (Berson et al., 2002; Do, 2019; Aranda and Schmidt, 2020). Moreover, ipRGC dysfunction can disclose and verify ocular and neurologic diseases, such as glaucoma (Rukmini et al., 2019). Initial animal research demonstrated the potential utility of ipRGC driven gene therapy to preserve and/or arrest vision loss in blinding diseases (De Silva et al., 2017). In our prior research we used selective chromatic adaptation, comparable to short-wavelength automated perimetry (SWAP), wherein a blue stimulus on a very bright amber background suppresses input from rods, green (M) and red (L) sensitive cones, allowing quantification from short wavelength sensitive neurons including S cones and ipRGCs. By using a relatively long duration stimulus we quantified S cone responses as well as putative retinal (ERG) and cortical (VEP) responses from ipRGCs (Rabin et al., 2021). Herein selective chromatic adaptation was coupled with a blue (S) cone blocking filter (Rosco GamColor #480), to measure putative visual thresholds in response to selective stimulation of the ipRGC retino-cortical pathway using the Full-field Stimulus Threshold test (Diagnosys*FST*^®^, Diagnosys, LLC). The purpose was to develop an expedient technique to quantify large field ipRGC psychophysical thresholds. Potential applications include disease detection and monitoring, gene therapy diagnostics and efficacy, and as an outcome metric in clinical trials.

## Methods

### Isolation of S cone responses

**FIGURE 1:**
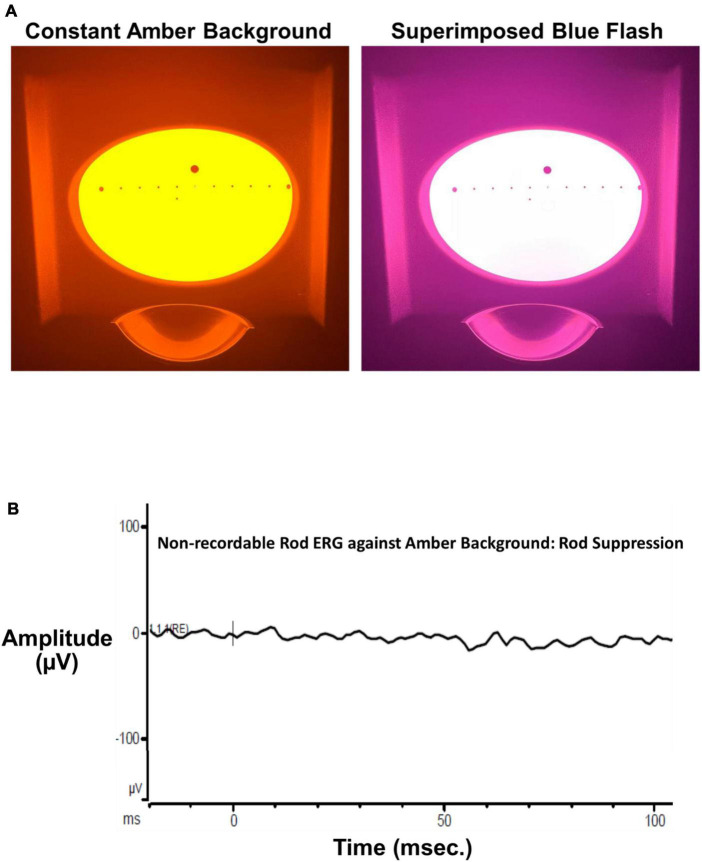
**(A)** The left panel shows the Ganzfeld stimulus illuminated by the bright amber L, M cone, and rod suppressing background (590 nm, 560 cd/m^2^ Diagnosys, LLC). The right panel shows the same Ganzfeld with the superimposed 200 ms blue flash (460 nm dominant wavelength). **(B)** The ISCEV standard scotopic (rod) ERG flash (4 ms, 0.01 cd⋅s/m^2^) superimposed on the constant amber background. The ERG was non-recordable from a visually normal subject after 30 s adaptation to the constant background verifying a lack of rod input to thresholds measured in the present study.

A Ganzfeld stimulator (ColorDome™, Diagnosys, LLC, Figure 1A), used for full field retinal stimulation including electroretinograms (ERGs), presented blue flashes (462 nm LED peak based on measured CIE chromaticity*: x, y* = 0.141, 0.027, full width at half maximum: 30 nm) on a constant amber background (590 nm, 560 cd/m^2^) to suppress input from L and M cones and rod photoreceptors. Luminance and chromaticity of stimulus and background adhered to international standards to isolate S cones for full-field ERGs (International Society for Clinical Electrophysiology of Vision, ISCEV)^1^ (Perlman et al., 2020). Moreover, the amber field adaptation time and luminance were derived directly from the Diagnosys, LLC S cone ERG protocol and consistent with the published ISCEV S cone ERG testing standard (Perlman et al., 2020). The luminance contrast of the threshold blue stimulus on the amber background in this study, which depends on L and M cone stimulation, was 0.003%, well below the psychophysical threshold for luminance contrast (Campbell and Green, 1965). This indicates that the contrast of the blue on amber stimulus was too low to stimulate L and M cones, consistent with our prior study of S cone and ipRGC ERGs and VEPs which revealed no L, M or rod contributions to the complex waveforms (Rabin et al., 2021). Finally, as stated in our prior study (Rabin et al., 2021), presentation of the ISCEV standard scotopic ERG flash stimulus against our amber background produced no recordable ERG substantiating the lack of rod input (Figure 1B).

### Isolation of IPRGC responses

**FIGURE 2:**
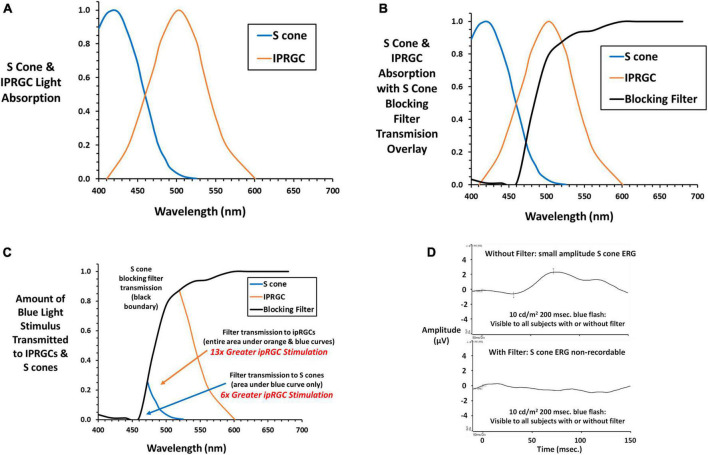
**(A)** Normalized absorption curves are plotted against wavelength for S cones (cyanolabe see text footnote 2) and ipRGCs (melanopsin) (Allen et al., 2019) photopigments. **(B)** The transmission curve for the S cone blocking filter (SCBF, Rosco GamColor 480 medium yellow) is overlayed on the S cone and ipRGC absorption curves. **(C)** The amount of light transmitted to S cones and ipRGCs is illustrated. The small area with blue boundary is transmitted to S cones, while this area plus the larger area with orange boundary is light transmitted to ipRGCs. By integrating under the curves, light transmitted to ipRGCs is 6 × greater than S cones within the S cone area, and 13 × greater to ipRGCs under its entire curve exemplifying greater stimulation of ipRGCs through the SCBF. **(D)** The S cone onset ERG (Rabin et al., 2021) to a long duration blue light stimulus (10 cd/m^2^) 10 × higher than stimuli presented during threshold testing shows a small but definitive S cone onset ERG with no recordable ERG with the SCBF in place substantiating the absence of S cone input. Please see text for further details.

The S cone blocking filter (SCBF, Rosco GamColor #480) was designed to minimize stimulation of S cones while retaining adequate stimulation of ipRGCs. It was secured before the subject’s eyes using a wrap-around lightweight plastic frame with an adjustable strap to minimize vertex distance and maximize field of view. Figure 2A shows normalized absorption curves for S cones^2^ and the ipRGC photopigment melanopsin (Allen et al., 2019) exemplifying longer wavelength blue light absorption by ipRGCs compared to S cones. Figure 2B shows the same curves with an overlay of light transmitted by the SCBF filter. The impact of the SCBF is better exemplified in Figure 2C showing light transmitted by the filter to S cones compared to ipRGCs. The small area with blue boundary is light transmitted to S cones while this area plus the larger orange bounded area is light transmitted to ipRGCs. Integration under the curves revealed a 6 × greater stimulation of ipRGCs in the blue cone area and 13 × greater ipRGC stimulation overall. To better quantify relative stimulation of S cones vs. ipRGCs through the SCBF, normalized stimulation was calculated at the wavelength upper limit of 1.5 × the blue LED full width at half maximum (484.5 nm). Stimulation of ipRGCs (0.54) was 4.5 × greater than S cone stimulation (0.12) at this wavelength. Moreover, for mean thresholds obtained in this study, computation of S cone excitation (E) and conversion to S cone Weber contrast: (Rabin, 1996; Rabin et al., 2011)


(SConeE-stimulusandbackgroundSConeE)background/

$$S\mathrm{Cone}E{}_{\text{background}}$$

revealed a 65 × decrease in S cone contrast when the stimulus was presented through the SCBF compared to no filter. As in the present study, in our previous study of S cone and ipRGC ERGs and VEPs, we used a long duration blue light flash (200 ms) to capture both on and off responses and the longer latency ipRGC signals. Figure 2D shows the onset portion of the S cone ERG elicited by a 10 cd/m^2^ 200 ms flash visible with and without the SCBF. Note that ERGs are typically recorded with a short duration flash (≤4 ms) and expressed as cd⋅s/m^2^ with the Diagnosys LLC protocol using a 4 ms 1 cd⋅s/m^2^ flash. Conversion of our 10 cd/m^2^ stimulus to short flash units yields a much dimmer 0.04 cd⋅s/m^2^ stimulus which accounts for the small amplitude S cone ERG. Nevertheless, it was non-recordable (flat ERG) through the SCBF confirming minimal S cone stimulation. The same result was obtained with the highest luminance long duration blue flash used in our prior study and the maximum achieved with our system (16.7 cd/m^2^). Finally, when viewed through SCBF, the luminance contrast of the threshold blue stimulus on the amber background was 0.15%, well below the psychophysical threshold for luminance contrast (Campbell and Green, 1965) indicating no intrusion from L or M cones through the SCBF.

### Threshold methodology

**FIGURE 3:**
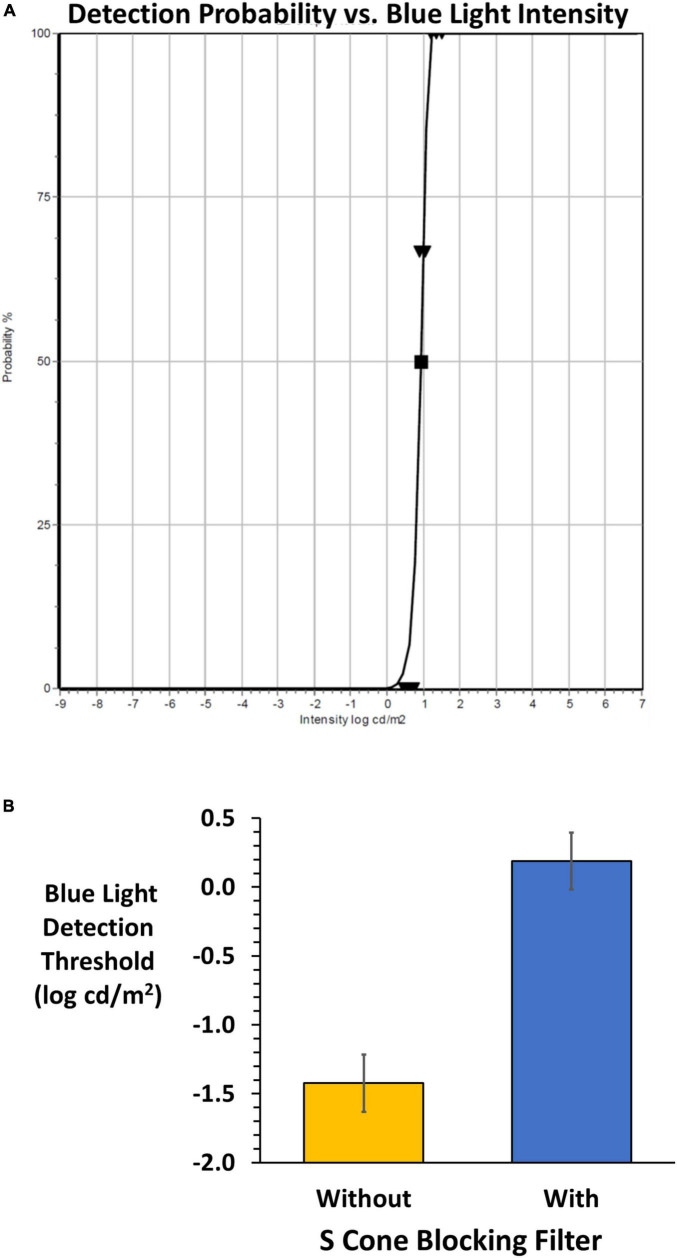
**(A)** An example of the FST^®^ result from the adaptive staircase for a subject wearing the SCBF. Validity is high (grade 3) based on 0 and 100% responses, slope and deviations from the fitted Weibull function yielding a 50% detection threshold (log cd/m^2^). **(B)** Mean (± 2 SE) FST^®^ 50% detection thresholds without (orange) and with (blue) the SCBF. Lower thresholds indicate higher sensitivity, and higher thresholds lower sensitivity. With the SCBF stimulation was limited to ipRGCs and the difference between S cone and putative ipRGC thresholds is highly significant (*P* < 0.001).

The Diagnosys*FST*^®^ (FST^®^, Diagnosys, LLC) uses a probability density Weibull distribution to determine the light detection threshold seen 50% of the time. The FST^®^ was modified to allow presentation of the blue stimulus on the amber background. The subject uses a yes-no detection device to input responses during the adaptive staircase. The device is a hand-held two-button box to report detection (depress right green button) or non-detection (depress left red button). A training session was given prior to measurements. Each test commenced with 30 s adaptation to the amber field followed by 200 ms presentations of the blue stimulus presented against the constant amber background. A beep signaled each stimulus onset, and the subject was allowed 3 s to respond. Testing was conducted on right, left and both eyes with and without viewing through the SCBF in randomized order. This preliminary report details monocular results from subjects who showed high validity based on software quality scores (0–3). High quality scores (2 and 3 in our sample) were based on the number of 100 and 0% detections, deviations from the fitted functions to determine threshold as well as the function slopes. Figure 3A shows typical results when viewing through SCBF (high quality score: 3).

Nineteen healthy young adults (mean age ± SD: 30 years old ± 10, range: 18–45 YO) participated after providing written informed consent in accord with our IRB approved protocol and the Declaration of Helsinki and its revisions. Data analyses were conducted with Microsoft Excel (version 2211). Thresholds were distributed normally (Jarque-Bera test). Two-way repeated measures ANOVA was used to compare data across filter vs. no filter and right and left eyes, with *post hoc* two-tailed *t*-tests with Bonferroni correction for multiple comparisons to confirm individual differences. Regression and Bland Altman analyses were used to substantiate data validity.

## Results

FST^®^ thresholds were significantly higher with the SCBF compared to without (*F* = 418, *P* < 0.001) with no difference between right and left eyes with or without the filter (*F* = 0.01, *P* > 0.94). Therefore, mean thresholds of right and left eyes were used for analyses. Mean threshold with the SCBF filter (0.19 log cd/m^2^) was significantly higher than without the SCBF (−1.42 log cd/m^2^, mean difference 1.61 log cd/m^2^, 95% CI: 1.38–1.84, *P* < 0.001, Figure 3B).

**FIGURE 4:**
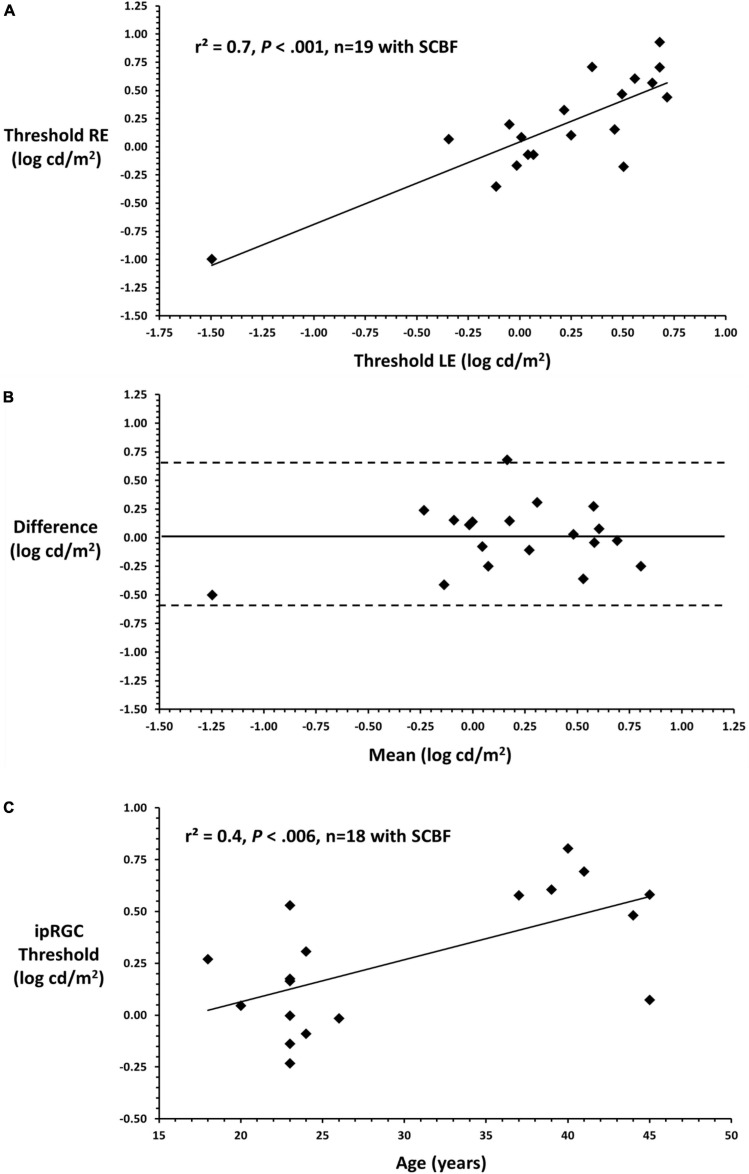
**(A)** Linear regression between right and left eyes through the SCBF (putative ipRGC thresholds) shows a significant relationship between eyes substantiating the validity of this metric in subjects yielding high quality scores. **(B)** Bland-Altman plot comparing right and left eye putative ipRGC thresholds shows no significant interocular bias (mean difference 0.1) which approximates 0 and no bias. All data fall within 2SDs of the mean difference between eyes substantiating validity and reliability of measures from this visually normal sample. **(C)** Mean ipRGC thresholds from right and left eyes are plotted against age.

To illustrate the variability of putative full-field thresholds from the ipRGC retino-cortical pathway and correlation between eyes, linear regression was conducted between right and left eyes on data recorded through the SCBF (Figure 4A). Data from right and left eyes showed high predictability (*F* = 39.0, *P* < 0.001, *r*^2^ = 0.7). Bland-Altman analysis between right and left eyes showed no inter-ocular bias (mean interocular difference = 0.1, Figure 4B) with thresholds within 95% confidence intervals substantiating validity of measurements with selective chromatic adaption through the SCBF. Insofar as there was no difference between right and left eyes, an initial estimate of the coefficient of repeatability (COR), which is the 95% confidence interval for within subject change, was computed from the standard deviation of the differences between right and left eyes multiplied by 1.96 (Bailey et al., 1991). This yielded a COR of 0.6 log units indicating that for this initial sample, a within subject difference of 0.6 log cd/m^2^ indicates a significant change in subjects or patients over time, an important metric for clinical applications. Finally, there was a significant correlation between putative ipRGC thresholds and age (*r*^2^ = 0.4, F = 10.5, *P* < 0.006, Figure 4C), consistent with recent reports (Esquiva et al., 2017; La Morgia et al., 2017; Spitschan et al., 2017). For this initial age assessment, data from one young subject with a low threshold was not included as a possible outlier. The preliminary age effect further substantiates the validity of this new method since age effects even between 40 and 50 may reflect the limited number/lack of redundancy in the ipRGC pathway.

## Discussion

This study describes a rapid, clinically expedient technique to obtain putative full-field thresholds from the ipRGC retino-cortical pathway. The combination of the time-honored technique of selective chromatic adaptation, wherein a bright amber field suppressed input from L and M cones and rods, combined with an S cone blocking filter, enabled quantification of putative large-field visual thresholds from ipRGCs within 2–3 min.

Study limitations include the availability of the combination of selective chromatic adaptation and the SCBF to isolate ipRGC thresholds. However, both can be made available with the approach described herein or a similar paradigm. Moreover, this approach may be less susceptible to intrusion from other receptors and pathways when ipRGCs are isolated using silent substitution which typically is conducted at higher contrast and requires very exacting chromaticity (Rukmini et al., 2019; Conus and Geiser, 2020). It is also possible that thresholds derived when viewing through the SCBF may reflect input from S cones, but the limited filter transmission of the blue stimulus to S cones, and the significant decrease in S cone excitation and contrast through the filter make this unlikely. However, it is critical to note that, in the current paradigm, the combination of blue stimulus and blocking filter also decreased stimulation of ipRGCs. Hence, while useful, the putative thresholds reported herein should not be considered absolute measures of ipRGC sensitivity. Potential improvements may be achieved by limiting the filter to the LED light source or possibly utilizing a green LED within the absorption spectrum of melanopsin.

Several exceptional studies have reported human visual responses mediated by the ipRGC retino-cortical pathway (Brown et al., 2012; Spitschan et al., 2017; Allen et al., 2019; Lucas et al., 2020). All studies used silent substitution methods to isolate ipRGC responses by selectively stimulating ipRGCs based on their spectral sensitivity while “silencing” input from conventional receptors by choosing metameres which have minimal difference in stimulation to each individual receptor (L, M, S cones, and rods) similar to recent FDA approved cone specific color vision tests to isolate cone contrast sensitivity (Rabin, 1996; Rabin et al., 2011). While the majority of ipRGC studies used large stimulation fields, Allen et al. (2019) used centrally viewed sinusoidal gratings to determine contrast thresholds mediated by ipRGCs, emphasizing their low spatial and temporal selectivity which is seminal for our understanding. Spitschan et al. (2017) added fMRI to support the validity of their larger field findings. Notwithstanding the innovation, comprehensiveness and excellence of this research, use of multiple techniques to isolate ipRGCs using silent substitution remains susceptible to inadvertent stimulation of conventional receptors particularly at high luminance and contrast levels. This suggests that more standardized methods are needed, perhaps a validated clinical test which could be more widely available (Conus and Geiser, 2020).

Herein we report isolation of ipRGCs thresholds using selective chromatic adaption to render L and M cones incapable of responding combined with an efficacious S cone blocking filter perhaps less susceptible to unintended intrusion from non-targeted receptors. The technique is specific to this study but could be standardized using the FST^®^ system combined with the SCBF. The importance of this preliminary study is exemplified by the myriad visual and non-visual functions mediated by ipRGCs including photo-entrainment and circadian rhythms, awareness, cognition, mood, pupillary light reflexes and conscious visual perception. Hence their role has assumed increasing importance for detection, diagnosis and monitoring abnormalities in various occupations including shift work, as well as acquired and traumatic brain injury, cognitive impairment from early onset and senescent disease including Parkinson and Alzheimer, as well as a host of ocular diseases impacting inner (e.g., glaucoma) (Rukmini et al., 2019) vs. outer retinal function (rod and cone dystrophies, age-related macular degeneration). Equally important, the exponential increase in gene therapy to arrest or improve hereditary retinal and optic nerve disease mandates sensitive metrics of full-field retinal stimulation as reported herein (De Silva et al., 2017). We fervently hope this initial research will ultimately prove useful for diagnosis, detection and monitoring of visual and non-visual conditions and expand our understanding of ipRGCs.

## Data availability statement

The raw data supporting the conclusions of this article will be made available by the authors upon reasonable request.

## Ethics statement

The studies involving human participants were reviewed and approved by UIW the Human Subjects Institutional Review Board. The patients/participants provided their written informed consent to participate in this study.

## Author contributions

JR was responsible for conceptualization, conducting the investigation, formal analysis, writing, reviewing, editing the manuscript, and project administration. EP was responsible for conducting the investigation, formal analysis, and project administration. WP, GK, KH, VS, BA, and RS were responsible for data collection and curation. All authors contributed to the article and approved the submitted version.

## Footnotes

1
www.iscev.org

2
www.cvrl.com
